# A Machine Learning Method for Vision-Based Unmanned Aerial Vehicle Systems to Understand Unknown Environments

**DOI:** 10.3390/s20113245

**Published:** 2020-06-07

**Authors:** Tianyao Zhang, Xiaoguang Hu, Jin Xiao, Guofeng Zhang

**Affiliations:** 1 School of Automation Science and Electrical Engineering, Beihang University, Beijing 100191, China; tianyao@buaa.edu.cn (T.Z.); xiaoguang@buaa.edu.cn (X.H.); gfzhang@buaa.edu.cn (G.Z.); 2 ShenYuan Honors College of Beihang University, Beihang University, Beijing 100191, China

**Keywords:** UAV, visual RGB, real-time, YOLOv3, color detection, object detection, machine learning system

## Abstract

What makes unmanned aerial vehicles (UAVs) intelligent is their capability of sensing and understanding new unknown environments. Some studies utilize computer vision algorithms like Visual Simultaneous Localization and Mapping (VSLAM) and Visual Odometry (VO) to sense the environment for pose estimation, obstacles avoidance and visual servoing. However, understanding the new environment (i.e., make the UAV recognize generic objects) is still an essential scientific problem that lacks a solution. Therefore, this paper takes a step to understand the items in an unknown environment. The aim of this research is to enable the UAV with basic understanding capability for a high-level UAV flock application in the future. Specially, firstly, the proposed understanding method combines machine learning and traditional algorithm to understand the unknown environment through RGB images; secondly, the You Only Look Once (YOLO) object detection system is integrated (based on TensorFlow) in a smartphone to perceive the position and category of 80 classes of objects in the images; thirdly, the method makes the UAV more intelligent and liberates the operator from labor; fourthly, detection accuracy and latency in working condition are quantitatively evaluated, and properties of generality (can be used in various platforms), transportability (easily deployed from one platform to another) and scalability (easily updated and maintained) for UAV flocks are qualitatively discussed. The experiments suggest that the method has enough accuracy to recognize various objects with high computational speed, and excellent properties of generality, transportability and scalability.

## 1. Introduction

As humans, we can explore a new unknown environment with our eyes before taking actions and making decisions and so should UAVs (unmanned aerial vehicles). Backed up with the VSLAM, VO and optic flow techniques, the UAV can estimate its self-position on a calculated map, the size and position of obstacles based on disparity images, and its distance to the obstacles. However, this is not intelligent enough for the UAV. How many items are there in the environment? Are there any dangerous items in the environment? Is the security situation safe enough for the planned action? These questions require understanding ability from an intelligent UAV. Recognizing the items in an unknown environment is a basic ability when the UAV is executing a mission.

**Figure 1:**
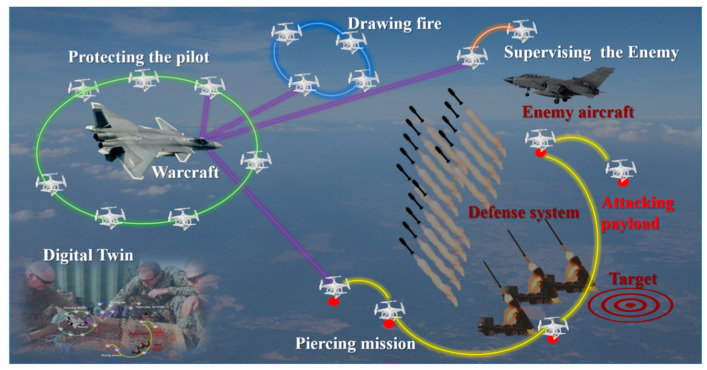
The application and necessary of understanding the environment. In a future war, the capacity of understanding unknown and complex environments would allow a UAV flock to complete more powerful missions.

The purposes of this study are investigating a method to equip UAVs with recognition capacity and evaluating the impact of the method on the real time performance and the detection accuracy under working conditions. For practicability purposes, the method is integrated in a UAV system which has the potential of managing several UAVs. This paves the way to making the UAV or the UAV flock intelligent and this UAV system could be used widely including in military applications, as shown in Figure 1, indoors and outdoors search and rescue and transporting supplies in an urban scenario.

This research has practical value because the applications of UAVs have increased dramatically, including infrastructure monitoring [1], providing bird’s-eye-view images and a communication network for rescuers and transporting medical supplies [2]. Images from UAVs flying a few meters above the ground fill a gap between the costly low-resolution images provided by satellites and narrow view images provided by fixed monitors.

Liberating the labor force is another scientific contribution of the method proposed. Kolling et al. [3] illustrate that the operator could not operate more than four UAVs at the same time, due to the cognitive complexity of human-robot systems, while as shown in Figure 1, the man-UAV system might contain far more than four UAVs in the future. As mentioned by Hocraffer et al. [4], currently, most single UAVs use manual operations, which is the lowest Level of Automation (LOA); the other three levels are the human-oriented semi-autonomy, the machine-oriented semi-autonomy, and the fully autonomous [5]. Research has found that as automation increases, operators could effectively guide or direct UAVs in more complicated tasks [5]. The purpose of the proposed method is to equip UAVs with artificial intelligence to understand the environment and facilitate human convenience.

Supported by the camera-equipped UAV, humans could complete challenging tasks unimaginable before. Militarily, solider could decrease the risk of wounds and injury by spying aerially on the enemy remotely before acting [6]. Commercially, humans could record a video from various viewpoints, quickly transport objects regardless of restrictions on traversable roads, and aerially track targets of interest for a long time [2]. With the further development of UAVs, teams of UAVs (a UAV flock can consist of hundreds of UAVs) could play important roles in future applications.

An application of a UAV system is shown in Figure 1, which also illustrates the necessary of understanding unknown environments. The quantity of aircraft in a UAV system can vary. The man in the UAV system has the highest priority, but does not operates certain UAVs directly. As shown in Figure 1, the missions of UAVs vary from group to group, including protecting the pilot, drawing fire from the enemy, piercing defense systems, and surveilling the enemy. The foundation of these high-level capabilities is the understanding of generic objects in real time. Therefore, this paper proposes a real-time understanding method, which combines machine learning technology and traditional image processing, to detect generic objects. Although the example here is related to military applications, the scientific contribution of this example could be easily adopted to civilian use, like assisting rescuer for search and rescue.

In this paper, the information of the environment is represented as the object category, location of the object in the image, and the color blob of the object in the environment. The environment is unknown since there is not information about the objects, marks, dynamics and scope. This environment could be an indoor or outdoor environment, and the application of a UAV is not limited to the urban scenario. The object is not always below the UAV. Thus, the images should not always be taken from a bird’s eye view. This constraint is meaningful and makes the system easily extensible to practical applications. This paper takes a rotorcraft, like a quadrotor, as an example for the convenience of experimentation.

The representation of the environment could be extracted based on computer vision technology [7], including the semantic segments, depth perception, object classes, room layouts and scene class [8]. However, most of the high level representation methods mentioned above require costly and high performance computational resources. Recently, to build small and low latency models that are matched the requirements for mobile and embedded vision application is still a research hotspot [9,10,11]. In other words, equipping the UAV which has limited computational resources with the ability of understanding an unknown environment through images remains a serious challenge.

We use cameras to understand the environment and detect generic objects in the environment, considering the cost, computational efficiency, weight, power dissipation and the information extracted. A video could be processed as a series of sequential images. Hence, RGB images in this paper represent the frames of video captured by the UAV. The UAVs are always equipped with multiple integrated sensors for understanding, mainly through radar and/or electro-optical/thermal (EO-IR) sensors and less commonly through acoustic and radio frequency (RF) sensors [12]. Compared with other types electro-optical sensors (like cameras) are cheap, sensitive to environmental settings, and can detect and classify with the highest capability if the target is visible. Images do not require energy to interrogate the environment and can gather richer information and span wider fields of view, compared with other distance sensors such as sonar, infrared and laser range finders.

We utilize a machine learning method, YOLO, and a traditional method, color blob detection, to understand the environment. Visual Simultaneous Localization and Mapping (VSLAM) algorithms are also a vision-based approach for understanding the environment method but these were not studied in this paper. VSLAM aims to construct a consistent map of the environment and simultaneously estimate the global position of the robot within this map [13]. Visual odometry (VO) algorithms handle the problem of estimating the 3D position and orientation of a vehicle [13]. Although VSLAM and VO are precise pose estimation methods, their limitation is clearly that they are not the methods for recognizing the category of objects in an unknown environment.

The method proposed in this paper makes full use of the development of computer vision technology and hardware platforms to understanding. We analyzed the detection accuracy with the mean Average precision (mAP) metric. Our experiments demonstrate that the UAV could be armed with state-of-the-art computer vision algorithm, the detection accuracy in working condition is sufficient and the real-time performance is still acceptable.

The real-time performance here is restricted to capturing and processing images and providing mission feedback during the execution of the mission. The mission of an UAV, like searching for a target, could respond to the processing result. We address the understanding method with this property as onlined in this paper. In contrast, the understanding method to extract the information is offline if the UAV has already finished the aerial mission and the processing result has no influence on the mission (e.g., filming an area [14]). The online method to extract the information from real-time images has wider application prospects than the offline one. Thus, abundant research, including ours, focus on online methods run on an embedded system [7,15].

The design of the UAV system should consider human-robot interactions and maintenance of the system, when the quantity of UAVs increases. This paper takes the interaction between humans and UAVs into consideration and implements a closed processing cycle of images from capturing and processing images to displaying them and decision making. For a large-scale use of the method in an UAV flock, generality (can be used on various platforms), transportability (easily deployed from one platform to another) and scalability (easily updated and maintained) are essential for the maintenance of the system. With this method, we have participated in MISSION 8 of the International Aerial Robotics Competition (IARC), 2018. The system won the first prize and the Best Man-Machine Team award in the competition, which also, to some extent, proves the practical value of the proposed method.

Furthermore, to evaluate the detection accuracy of the UAV system, we randomly sampled the COCO dataset. We evaluated that by sending the results collected from the smartphone to the server program (provided by COCO). That means our detection accuracy is determined by the performance of the YOLO model on the smartphone under working conditions. Since the execution platform is a smartphone with limited memory for datasets and computational resources for detection, the analysis of evaluation results and the comparison with other computer vision algorithms are rough, however, still valuable in practice.

To summarize, considering the above factors, the contributions of this paper are fourfold: (1) A novel understanding method is presented. We propose an understanding method, which combines machine learning and traditional image processing algorithms, for UAVs to sense 80 classes of objects (listed in Appendix A) in an unknown environment through real-time RGB images; (2) Liberation of the labor force. Detecting generic objects increases the intelligence of UAV and decreases the need of a labor force for operating the UAV, searching for target in the video, and triggering the next mission. This also serves as preparation for the large-scale use of UAVs in UAV swarms; (3) Prototype verification. We implement a closed image cycle from capturing and processing to displaying and decision making, utilizing semi-physical simulations and physical experiment to evaluate the properties of the approach. (4) We quantitatively evaluate the impact on the real time performance and the detection accuracy under working conditions, using frames per second (FPS) to evaluate the real time performance using mAP metrics and the randomly sampled COCO dataset (10,000 images with 80 categories) to evaluate the detection accuracy of the method.

## 2. Related Work

This section describes a comparison with other solutions of the problem of equipping the UAV with the capability of understanding an unknown environment.

### 2.1. Methods for Understanding the Environment

Researchers have expended a great deal of effort in implementing visual ability in UAVs. Some methods use sensors working in an active way [16], such as synthetic aperture radar (SAR) or light detection and ranging (LIDAR). These methods can be easily used during cloud cover, night, and rain conditions, which makes the method independent of the weather conditions. However, these methods need more energy to interrogate the environment than RGB images, which limits the cruising ability of the UAV.

Some methods use sensors in a passive way, such as event-based cameras [17], RGB-D cameras, and RGB cameras. These sensors are light in weight and rich in information. Falanga et al. [17] used event-based cameras to achieve outdoor dynamic obstacle avoidance capability but the cost of the event-based camera is too high and it could not recognize generic items. Using RGB-D camera, researchers could generate a global map and compute the location easily, but these visual SLAM and VO methods require powerful computational capability, which is not suitable for UAVs with limited resources. Using a RGB camera, Blösch et al. [18] localized and navigated an UAV in an unknown environment with help of a parallel tracking and mapping (PTAM) algorithm. Although the real time performance was good, their method could not be used in our scenario because it needs a powerful ground station to run the program and it cannot recognize generic items in the environment.

Optic flow technology is one of the methods for implementing the visual ability of UAVs by using passive sensors [2]. This technology is inspired by the compound eyes of insects, which are dense arrays of facets pointing in different directions and spanning large fields of view [19]. However, it is difficult to maintain the hover mode for a long time due to the limitations of optic flow algorithms. Besides, optic flow only uses part of the information contained in RGB images, and this technology is sensitive to the light conditions.

Considering the cost, computational efficiency, weight, power dissipation and the information extracted, the method proposed in this paper only utilizes RGB images from RGB cameras, which not only provides abundant information, but also saves energy to enhance the UAVs cruising ability.

### 2.2. Detection Ability of UAVs

Recently, several studies have focused on the development of UAV imagery for engineering applications using artificial intelligence techniques (UAV-AI). Some studies endows the UAV with real-time visual ability to localize tree branch-like surfaces for perching [20]. This work focuses more on special object detection for UAVs. Zhou et al. [14] used an updated YOLO v3 model to detect the opium poppies in images captured by an UAV. Compared with original YOLO v3 model the model uses the recently proposed Generalized Intersection Over Union (GIOU as the loss function, and a Spatial Pyramid Pooling Unit is added [21], while, the method is run on a RTX2080Ti platform, which means the detection process is offline and could not benefit the automatic control immediately for UAVs in an unknown environment.

Gao et al. [22] built a system that can detect agricultural croplands and orchard areas by online and offline methods, which is not the same task definition as ours. The online method in [22] represents that learning and recognition are at the same time, and offline means the phases of learning and recognition are separated. Their algorithm is conducted on the ground station with the help of MATLAB to process the images which are collected from low (5 m) and high (15 m) altitude by the UAV. The UAV here is just a sensor used to collect the images above the ground for the system.

Some studies detect cooperative UAVs using a vision-based approach with the help of navigation data [7,23]. The system proposed by Opromolla et al. [7] finds out the search area in the picture, after getting the relative position through inter-vehicle data links. Then, it uses a template matching method with the help of a previously generated database to detect cooperative UAVs. Opromolla et al. [23] used YOLO v2 to replace the template matching method to find the bounding box. Their method was implemented on a Robot Operating System (ROS) and the frames per second are 1 Hz and 7 Hz. These methods need extra signals, like navigation data of the target, to enable the method. They assume that the target (target UAV) has a connection with the UAV (tracking UAV), but we do not. Mostly, navigation data of the target does not need to be collected. Our method only uses RGB images and is implemented on an Android system with a much higher frame rate of 18 Hz to 23 Hz.

An UAV system with online object detection capabilities that run on a smartphone is not a novel concept. In the work of Hummel et al. [24], the video captured by an UAV is displayed and processed on a smartphone for the operator, and commands could be sent from the operator to UAV. However, the image recognition in their article needs several assumptions, including the size and height of the recognized book in pixels, and the shape of the book should be pre-configured. In the work of Martinez et al. [25], they implemented YOLO v3 based on OpenCV library to recognize generic items, used the images transformed from YUV format (video compressed with H.264 protocol is transferred from UAV to a smartphone, and then decoded into YUV format, but the YOLO needs RGB format inputs), and analyzed the detection accuracy of the model on the computer not under working conditions. This approach is outstanding, but the computational efficiency could be improved and the detection accuracy could be analyzed more credibly.

Compared with their work, firstly, our method uses the computational resources of the UAV and could interact with the UAV to complete more complex tasks, which is far more than an image processing program. Secondly, our approach utilizes a state-of-the-art recognition algorithm without extra assumptions and could recognize 80 classes of objects. Thirdly, we use OpenCV and TensorFlow. That means our method is more functional with the potential of object detection, image segmentation, gesture classification, and pose inference. These functions are supported by TensorFlow and the computational efficiency is guaranteed by the TensorFlow which focuses more on deep neural network processing. Fourthly, we analyzed the detection accuracy by sending the results collected from the smartphone to the server (provided by COCO). That means our detection accuracy is the performance of the YOLO model on the smartphone under working conditions. Furthermore, we qualitatively analyzed the scalability to update the system with a new recognition algorithm and maintain the system with a custom recognition algorithm.

### 2.3. Machine Learning Algorithm for Detection

Object detection has attracted the interest of researchers for decades. Normally, in order to increase the computational efficiency, an image would be resized and filtered as a preprocessing step. Sometimes, transform algorithms are used to detect certain textures or color blobs. With the help of a Fast Fourier Transform (FFT) algorithm, it is easy and fast to transform the information from the time domain to the frequency domain to detect texture. With the help of transforming the value of color image from red, green, and blue (RGB) space into hue, saturation, value (HSV) space, the algorithm detects unique colors precisely.

Traditionally, template matching algorithms, which compare the similarity of the patch in the image and a template in a pre-generated database, are widely used [23,26]. However, their poor generalization ability limits the application of template matching algorithms. Template matching algorithms are suitable for applications of finding an object in the template using various stored views of the object. In our scenario, detecting 80 categories of objects needs a large number of templates which costs too much of the computation time.

Recently, as computational capability has increased, deep learning algorithms perform better and better. Especially after 2012, convolutional neural networks (CNN) have become the dominant method to extract the features of images in computer vision tasks, including image classification [27,28,29,30], object detection [31], and semantic segmentation [32]. Then the network goes deeper and deeper. The deeper the network is, the more parameters the network has, so the representation ability increases, and the network could discriminate subtle difference between different categories. However, the power consumption and sheer size of such models inhibit their practical use. Thus, researchers have investigated a lot of ways to break through this bottleneck.

One direction is quantizing and compressing the network [11]. The other direction is designing a new network architecture [9,10]. Both these approaches are effective and tested by practice. One example is the YOLO model, which is a novel architecture proposed by Redmon et al. [10,21]. There are three layers called YOLO layers, which represent three scales and contains three sets of information: (1) the coordinates of the bounding box; (2) the probability of it containing an object; (3) the probability of a certain category. The difference between each version is the network used for performing feature extraction: the first version uses 24 convolutional layers followed by two fully connected layers; the second version uses Darknet-19 and the third version uses Darknet-53 [21].

## 3. Materials and Methods

This section defines the goals of our research first, and then describes several basic terms to pave the way for our research.

### 3.1. Problem Formulation

In order to arm a man with an intelligent UAV for complex tasks, the primary challenge is to devise a scheme that equips the UAV with enough accuracy to recognize various objects online through RGB images, while not losing the performance features of generality, interactivity, transportability and scalability. To this end, this paper introduces an approach that utilizes a machine learning method to recognize 80 classes of objects through real-time video captured by an UAV. Furthermore, semi-physical simulations and physical prototyping verify the effectiveness of the proposed method.

### 3.2. Architecture of the System

**Figure 2:**
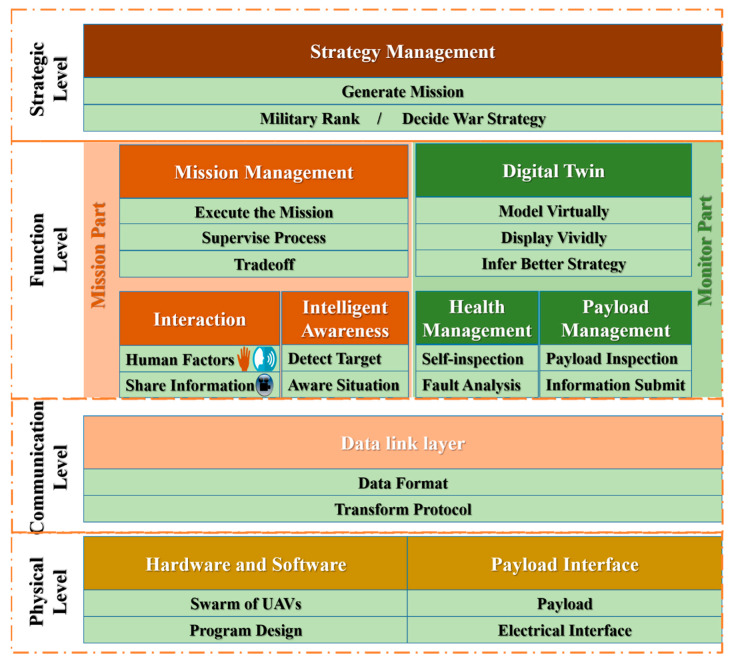
The architecture of UAV system. It is designed for the flock of UAVs and the overall system is composed of four levels: physical, communication, function, and strategy. This paper focuses on the Intelligent Awareness in the Function Level to empower the UAV with the capacity of understanding the environment.

The purpose of this part is the briefly introduce the UAV system that was equipped with the capacity of understanding an unknown environment. As shown in Figure 2, there are four levels and this architecture is designed for a flock of UAVs. The physical level includes the payload interface (like cameras and gimbals), the hardware (the UAVs) and the controlling programs. These are the basic parts of the system.

**Figure 3:**
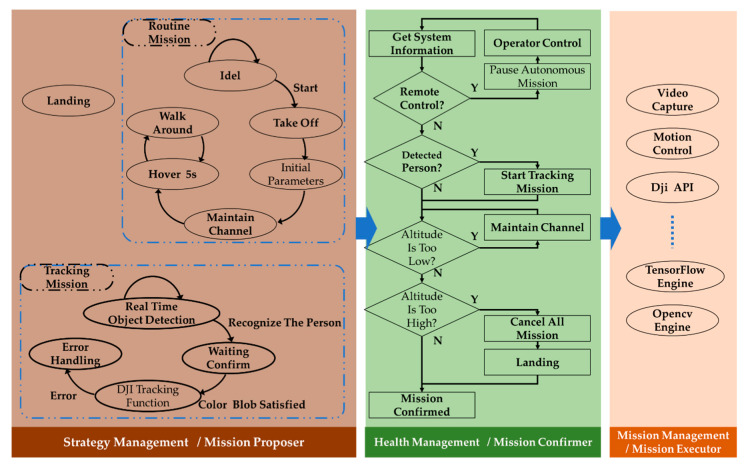
Software framework. In the program, the strategy management generates the missions and proposes one mission to be executed by the health management. The health management monitors the current system state and the security information, adjusts the mission if necessary and confirms it. The mission management would conduct the confirmed mission with the help of basic functions.

The communication is implemented at the communication level with the help of WiFi (connecting several smartphones, and the smartphones are connected to the remote controller by a USB cable) and radio (connecting the remote controller and the UAV). Here the video is transferred with the H.264 format from the UAV to the remote controller. The cooperation information is transferred based on the TCP/IP protocol from one smartphone to another. This communication level wil be described in detail in next section by the data flow. The mission part and the monitor part constitute the function level. Missions include take off, move forward, detect a target and so on. The interaction between the human operator and the UAV mission is discussed in a previous paper [33]. The solution and the performance evaluation of the intelligent awareness are studied in this paper. The monitor part takes care of the health of the UAV system. The strategic level is the highest level which generates a mission, delivers the mission and makes decisions. The strategic level is described in detail in Figure 3. Although it is verified by the practice, we so not analyze the performance and the efficiency of the architecture in this paper, and focus rather on the intelligent awareness function. The software framework is shown in Figure 3. For the convenience of organizing several functions and missions, we break the program into three parts: (1) Strategy management. This part collects missions and basic functions. In this research, there are two missions. The routine mission is an autonomous mission with a time sequence. After enabling the mission, the UAV takes off, initializes the parameters of the camera and gimble, adjusts the altitude to a certain flight channel [33] and then alternates between a hovering mission and the mission of moving around. This represents the behavior from taking off to searching autonomously. (2) Health management. This part checks the mission, which is proposed by the strategy management, and confirms the final mission to be executed based on the current system security and state. The health management monitors the system state every 0.05 s. (3) Mission management. In this part, the confirmed mission would be conducted.

### 3.3. Data Stream Pipeline

**Figure 4:**
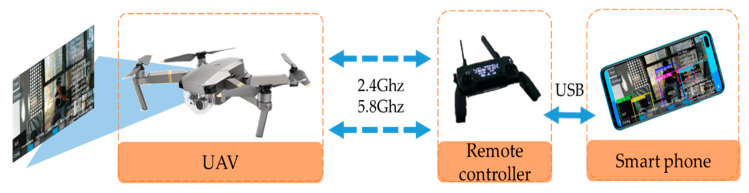
Data flow chart of the system.

To enhance the generality, interactivity, transportability and scalability from the mechanism, this paper introduces the framework of the method, and the data flow chart of the system is illustrated in Figure 4. As shown, the UAV senses the environment and records a color video with the first-person point of view (FPV). The video is encoded by the H.264 protocol (ITU-T Recommendation H.264. Standard for highly compressed digital video codec) and is transmitted to the corresponding remote controller through special electromagnetic waves.

After obtaining the video and flight information, the remote controller interacts with the smart phone through the Universal Serial Bus (USB) cable. The detected images in bitmap format are decoded from H.264. The main program to detect the object and real-time display is developed and deployed on a smartphone running the Android operating system. This program was developed in Android Java language based on the TensorFlow for Android library and OpenCV for Android library. The detection algorithm is packet as a “*.tflite”* file based on TensorFlow for Python rules.

As analyzed by the International Data Corporation (IDC), the Android operating system accounted for 87% of the market share in the smartphone domain in 2019. This ensures the generality of the proposed method and the prototype demonstrates the generality. Although the type and size of UAVs vary from task to task, the approach to transport the recognition method from one UAV to another just requires plugging the phone into another remote controller with subtle changes in the program. The scalability benefits from the design of the software.

### 3.4. Understanding Part of the Method

With the development of computer vision, lots of the representations $\left\{r^{1},r^{2},\cdots ,r^{n}\right\}$ could be extracted from the RGB images to sense an unknown environment. This paper takes three representations as an example, including the object class, location of the object in the image and color blob of the image.

**Figure 5:**
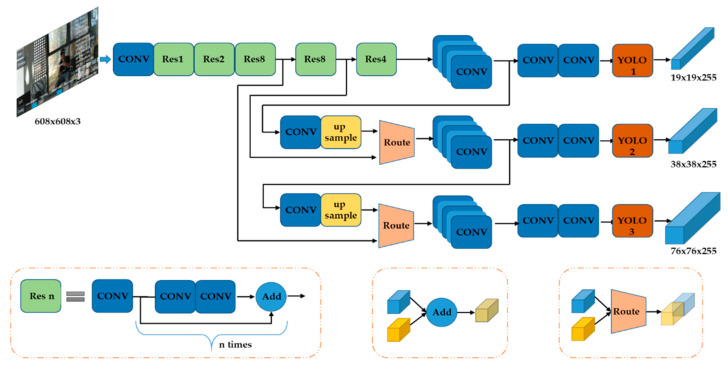
Structure of YOLOv3 used as recognition part of the method.

For representing the object class and location, this method utilizes YOLO v3 to recognize objects. The structure is illustrated in Figure 5. The resolution of the video is 1280 × 720 pixels. Thus, the size of the input layer in this network is configured as 608 × 608 × 3 (other options are 416 × 416 × 3, 320 × 320 × 3, 256 × 256 × 3 and smaller) to save more information when resizing. Normally, due to the limited computational resources, we resize the input images to 320 × 320 × 3. This would lead to narrowing the input layers. The network is 75 layers deep when counting only convolutional layers (or 107 layers if we count routes layer, shortcut layers, and YOLO layers). To determine the bounding box priors (means the location and the size of the target in the image), this network uses k-means clustering and chooses nine clusters with three scales. The nine clusters are: (10 × 13), (16 × 30), (33 × 23), (30 × 61), (62 × 45), (59 × 119), (116 × 90), (156 × 198) and (373 × 326) [21,24]. To extract the features Darknet-53 is used. The parameters are pre-trained by YOLO. With this configuration this model could recognize 80 classes of objects.

Next, to deploy the recognition algorithm on the smartphone, the model is optimized by using the TensorFlow Lite Optimizing Converter (TOCO) [34]. This step converts the resulting frozen graph into the TensorFlow Lite flatbuffer format. After the abovementioned process of the network, the category and the location of an object in the image could be figured out, and the object is covered by a bounding box.

In addition to the category of the object in an unknown environment, there is a lot of information encoded in the RGB images. Here we take the color blob detection as an example. Algorithm 1 illustrates the detection process.
**Algorithm 1** Color Blob Detection.**Require**: Real time RGB image;     the hue value of the ROI (region of interest);     the hue section1:**While** feed new frame **do**2: **if** processing **then** continue3: **else**4:   perform the down sampling step of the Gaussian pyramid construction5:   convert the value of RGB into the HSV color space6:   get binary image A(1 if the value is included in the hue section and 0 otherwise)7:   dilate the binary image A (morphological processing) into image B8:   retrieve contours from the binary image B using the algorithm Border Following9:   **if** the area of the region defined by the contour >50 pixels **then**10:     show the contour11:   **end if**12: **end if**13: process is finished14:**end while**

In order to blur an image and downsample it, the function performs the downsampling step of Gaussian pyramid construction. Before downsampling the image by rejecting even rows and columns, it convolves the source image with the kernel:

(1) $$K=\frac{1}{256}\left[\begin{array}{cc} \begin{array}{ccc} 1 & 4 & 6\\ 4 & 16 & 24\\ 6 & 24 & 36 \end{array} & \begin{array}{cc} 4 & 1\\ 16 & 4\\ 24 & 6 \end{array}\\ \begin{array}{ccc} 4 & 16 & 24\\ 1 & 4 & 6 \end{array} & \begin{array}{cc} 16 & 4\\ 4 & 1 \end{array} \end{array}\right]$$

The method to filter the noise is by dilating the image by using a specified structuring element that determines the shape of a pixel neighborhood over which the maximum is taken:

(2) $$\mathrm{imageB}\left(x,y\right)=max_{\left(x^{\prime },y^{\prime }\right):element\left(x^{\prime },y^{\prime }\right)\neq 0}\left\{\mathrm{imageA}\left(x+x^{\prime },y+y^{’}\right)\right\}$$

where imageB(x, y) represents the value of the imageB of x row and y column. And the $element\left(x^{\prime },y^{\prime }\right)$ here is the value of the 3×3 rectangular structuring element with $\left(x^{\prime },y^{\prime }\right)$ as the index of the row and column. To obtain the contours of the imageB, the well-known border following algorithm [35] is used. This algorithm is a topological structural analysis of digitized binary images.

### 3.5. Fusion at the Action Level

Understanding the environment is the preparation for the action. Therefore, this paper also investigated the action decision to evaluate the practicability of the proposed method. As a prior work [8] pointed out, fusion at the action level, which is predicting an action candidate from each representation and adaptively consolidating these action candidates into the final action, reduces redundancies and improves generalization.

Inspired by their approach, this paper proposes an alternative way of fusing the representations at the action level. At any time step t, when the system receives the RGB image input $o_{t}$, the proposed method computes the n representations for the image $\left\{r_{t}^{1},r_{t}^{2},\cdots ,r_{t}^{n}\right\}$. For each representation $r_{t}^{i}$, an action-prediction module $\pi_{i}(a|r_{t}^{i})$ produces an action candidate $a_{t}^{i}$, such as moving forward, avoiding the obstacle, and hovering. An n-dimensional vector $h_{t}=f\left(o_{t},M,C\right)$ is figured out by the function f, where M represents the mission goal and C represents the command from the operator. Then, $h_{t}$ is normalized with a softmax function to obtain the fusion weight $g_{t}=softmax\left(h_{t}\right)$. The final decision for the action $a_{t}$:

(3) $$a_{t}=\underset{i=1\cdots n}{\sum }g_{t}^{i}\pi_{i}(a|r_{t}^{i})\;,g_{t}=softmax\left(f\left(o_{t},M,C\right)\right)$$

The proposed method to sense the environment works well in the experiments with this fusion scheme.

## 4. Experiment Setup

### 4.1. Experimental Testbed

This paper conduct experiments using the DJI Mavic Pro and DJI Mavic Air UAVs (DJI, Shenzhen, China), which are consumer products with the technical parameters listed in Table 1. These products can avoid obstacles based on the distance calculated by the binocular camera and track objects based on interactions with the operator. One tracking mode is that the operator touch and drags a region of interest (ROI) on the screen and then the UAV could track an object in the ROI. Precisely, this is a tracking algorithm not a detection algorithm. Another tracking mode of the DJI is detecting a person (the only object) automatically and starting to track him. All in all, the built-in functions of the DJI UAVs cannot recognize various categories of objects. The more categories an UAV can detect, the more useful the system is for reducing the workforce.

As for the delay between when an object is visible to the camera and it is detected, this could be separated into two parts. One is the delay between when an object becomes visible and it is transferred to the smartphone. This delay is optimized by DJI, the UAV manufacturer, and is beyond the scope of this research. The video max bit rate is 100 Mbps, which means the delay of streaming images is so short that the operator should not feel it. The other is the delay of the model inference when the program runs under working conditions. This has the impact on the real time performance and is studied in this paper.

**Figure 6:**
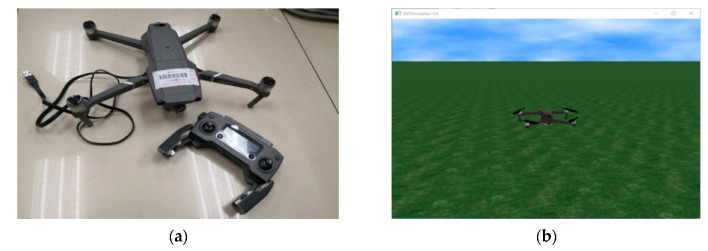
Semi-physical simulation system: (**a**) Physical part of the simulation; (**b**) Screenshot of the software.

The appearance of the DJI Mavic Pro is shown in Figure 6a. With the help of the on-board camera, the smartphone could obtain a video with a first-person point of view. The smartphone used in this paper is a Mate 10 Pro (Huawei, Shenzhen, China, with an octa-core CPU, Kirin 970 and 6 GB RAM). The purpose of using two UAV platforms is to evaluate the generality (can be used on various platforms), transportability (easily deployed from one platform to another) and scalability (easily updated and maintained).

As for the control algorithm and the algorithm for obstacle avoidance, we have published that in [33]. These algorithms have already been evaluated statistically in relation to performance, so this paper omits the performance of those algorithms. The machine learning method abovementioned and all the algorithms are implemented by Java and deployed on the smartphone. After being connected with the remote controller through a USB cable, the smartphone could control the motion of the UAV and receive data from the UAV. The operator can interact with the program at any time with the highest priority.

In the experiments, there are six states in the flight and four states in the test as shown in Table 2. One thing that should be emphasized is that this experiment only evaluates the real-time performance and the impact of the detection function on the flight control program. We chose three essential and vulnerable states to test the impact. The detection evaluation will be described later. This experiment works on the real time property of the understanding method in a different state. The understanding method includes two parts: color blob detection and object detection. IDLE state represents staying still on the ground with the motor powered off. The flight state would be changed automatically according to the time sequence as described in [33].

### 4.2. Semi-Physical Simulation Testbed

In addition to the prototype, a semi-physical simulation also is conducted for evaluating the proposed method. During the simulation, all the sensors including the camera work normally and that is the reason for the semi-physical simulation name. The purpose of the simulation is virtualizing the flight behavior to speed up the development progress and the detection accuracy evaluation in a safer way. After detecting the object through the camera in the real world, the mission command is computed, and the virtual UAV would move following the flight data from an inertial measurement unit (IMU).

This semi-physical simulation has two parts. First, the software on the computer is DJI Assistant 2 for Mavic, and Figure 6b shows a screenshot. Second, the physical part is the DJI Mavic Pro. The simulation environment is from DJI. During the simulation, the motors would be off and the IMU would keep working to send the computed flight information from the UAV to the computer through the USB cable. The simulation software would plot the flight trajectory on the screen.

Initially, we connect the UAV with a computer through the USB cable and turn on the battery. Then, open the software and run the simulation command. From now on, the commands from the program on the smartphone only will enable the IMU with the motors disabled. Flight data from IMU drives the virtual UAV in the simulation environment. This approach contributes to the development of the program in a safe way.

### 4.3. Detection Evaluation Setup

**Figure 7:**
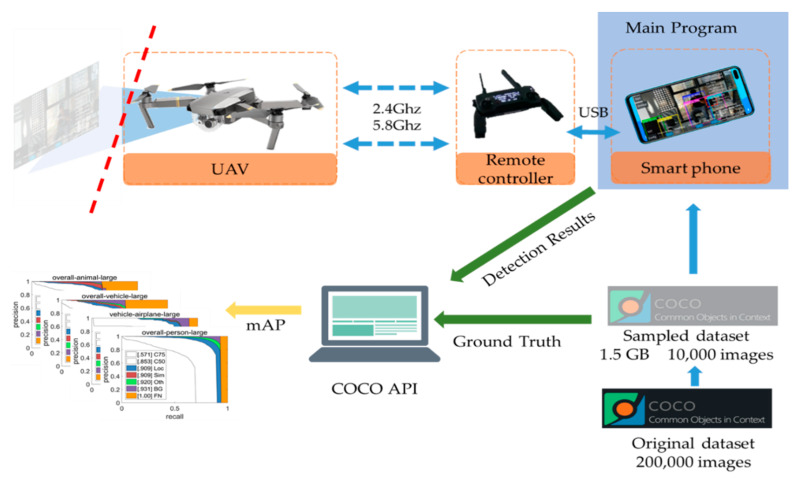
Detection evaluation setup overview.

To evaluate the detection accuracy, the famous COCO 2014 dataset (a publically available dataset) is used. One thing should be noted is that the purpose of the evaluation described here is verifying the detection ability of UAV system. We evaluate the accuracy based on the detection results computed on the smartphone under working conditions. Because of the memory limit of the smartphone which executes the program, we sampled randomly from the dataset and obtained 10,000 RGB images divided into 80 categories. The procedure takes nearly two hours to get 1.5 GB data from the COCO dataset, while transmitting the sampled dataset to a smartphone took more than six hours. An overview of the detection evaluation setup is shown in Figure 7.

The main program containing the detection algorithm is executed on the smartphone, and the images in the sampled dataset are loaded and detected by the phone. The detection results are in.json file format and are transmitted to the computer for analysis. The main purpose of this experiment is evaluating the detection accuracy. Thus, the image capturing function is disabled and the UAV is on the ground with the motor off but powered on. The application on the smartphone (the main program) is modified by adding a button for detection evaluation. The release version of the application does not have the button since the evaluation is not the main function of the proposed UAV system. This detection evaluation setup is reasonable since the only difference between the evaluation and a normal scenario is the image loading method. In a normal scenario, the images are captured by the UAV camera with a size of 1280 × 720, which contains more information than the images with the size of 320 × 320 employed in this evaluation scenario. That means the detection accuracy in the work scenario would be theoretically better.

**Figure 8:**
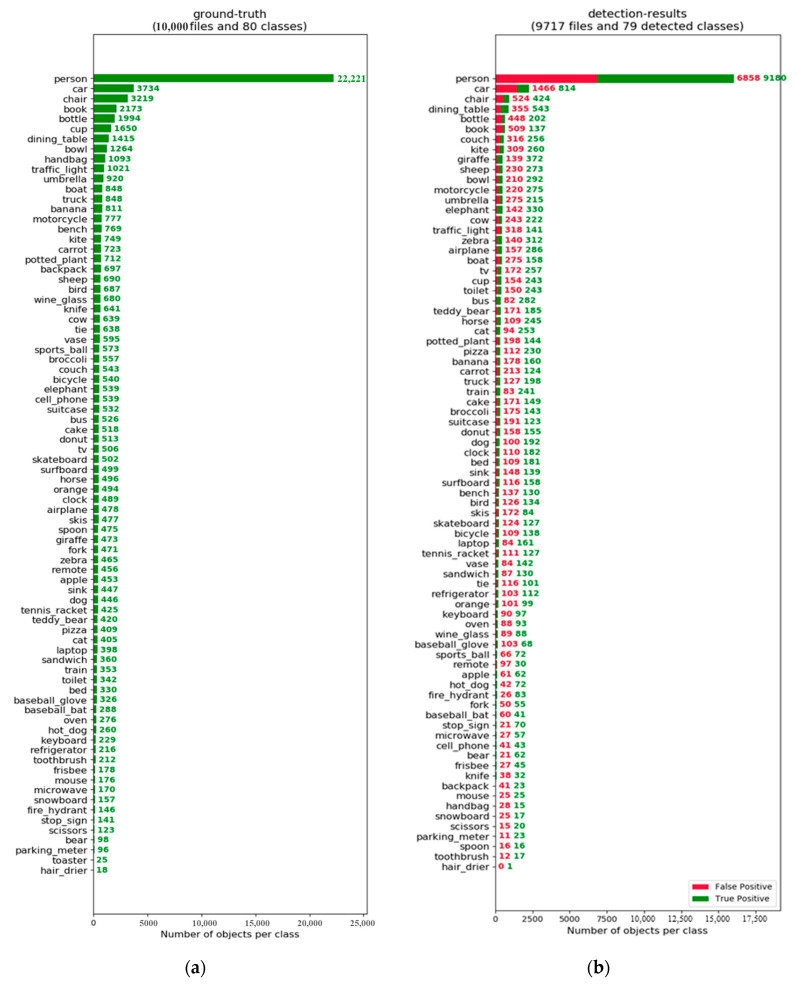
(**a**) Sampled dataset overview. (**b**) The detection results overview.

The number of images for each class is listed in Figure 8a. The number of images in the person class is more than 10,000 since lots of images contain more than five persons. The detection results of each class are shown in Figure 8b. There are 9717 images which are detected successfully, and the reason is discussed in the Results section. To evaluate the precision and the recall of the detection results, we used the standard COCO API tool provided by the COCO dataset.

### 4.4. Task Setups

For the convenience of investigating the generality, transportability and scalability of our proposed method, we select the task of tracking the person with a red helmet in an unknown environment. This task is an application test for the UAV system. This paper focuses on the evaluation of the method proposed for understanding the unknown environment, although the tracking capability of the Man-UAV system is also implemented. The real time performance is the main part of the investigation. In this task, the UAV autonomously flies in a random trajectory with a necessary collision avoidance algorithm. There are lots of persons in this unknown environment. The UAV should find the man with a red helmet, alert the operator, and then track this person. The video captured by the UAV would be processed and displayed on the screen for the operator and the operator could interact with the UAV at any time, like enabling the understanding method, confirming the target, and stopping the mission.

## 5. Results

### 5.1. Quantitative Evaluation

#### 5.1.1. Detection Accuracy Analysis

Detection accuracy is analyzed and discussed in this part first, and then some interesting results will be discussed. The analysis in this part is based on the sampled COCO dataset, and the detection results of images captured by the UAV lie in system analysis part. It takes 821 s from loading and detecting 10,000 images to storing the results in the smartphone. That means nearly 0.08 s for processing one image.

A comparison with other detection methods is shown in Table 3. On the one hand, due to the limitations of using a portable device, our results were done based on the selected 10,000 images which are less than the 20,000 images that others used for detection accuracy analysis. On the other hand, due to the limited computational resources, we resized the input images into 320 × 320 × 3, which results into information loss for a small object. Thus, the comparison analysis is rough. However, for the large object (area > 96^2^, where area is measured as the number of pixels in the segmentation mask), the AP (averaged across all 10 Intersection over Union (IoU) thresholds and all 80 categories) is 46.8, which is competitive with other methods.

**Figure 9:**
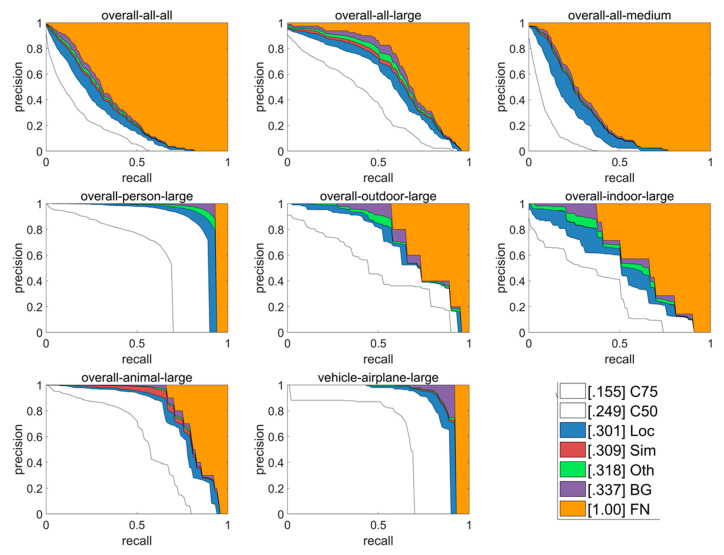
Detailed breakdown of false positives.

The detailed breakdown of false positives is shown in Figure 9. Large objects are detected much better than medium and small objects. Outdoor objects are detected more easily than indoors ones. Persons, animals and airplanes are well detected. Thus, our method has practical significance, to some extent, for an implementation of a sensing method for UAV systems.

**Figure 10:**
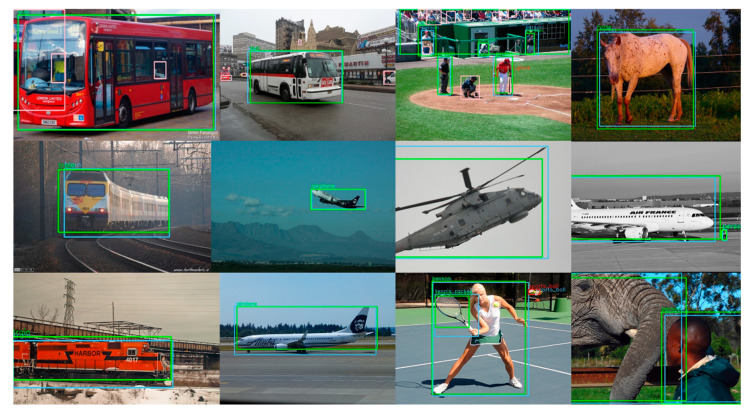
Several images that are successfully detected.

There are two issues that need to be discussed. The first one is the flight height of the UAV system. Due to the sensitivity to the object scale, the UAV height would be limited. Empirically, the height of 20 m is still acceptable. One solution to overcome this limitation is to adjust the focus of the camera, which is also supported on DJI Mavic Pro, to get a larger image from a longer distance, but this solution is not implemented in our recent system The second one is the influence of UAV vibration. The vibration has little impact on detection because of the gimbals mounted between the UAV and the camera. Several successfully detected results are shown in Figure 10. The green boxes are the detection results and the blue boxes are the artificial annotations of the images. We could find that the detection function works successfully.

**Figure 11:**
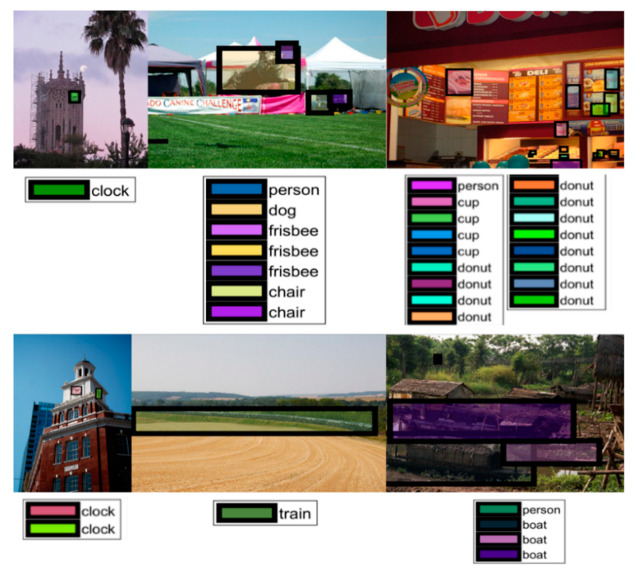
The ground truth of misdetections.

**Figure 12:**
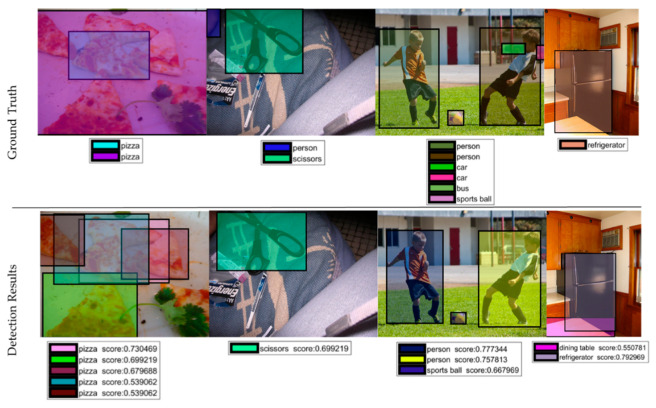
Some interesting detection results.

We went deep into the detection results to find the reason for misdetections and show several ground truths of the misdetected images in Figure 11. Honestly, it is also hard for humans to find the labels of the images. Small objects are hardly detected in Figure 11, which is agrees with the conclusion above. We also find some interesting results shown in Figure 12. First, some images are challenges for humans to find out what the ground truths are. Second, some images are detected successfully by the system, while the object is not labeled by the ground truth.

#### 5.1.2. Real-Time Performance Analysis

The real-time performance is influenced by the computational resources and the implementation method. Meanwhile, both the flight control and the environment understanding spend a lot of the computational resources of smartphones. Thus, it is meaningful to find a balance between controlling the flight and understanding the environment. Since the computational cost of flight control is various in different flight states, we analyzed the real-time performance across different flight states. The real time property that we focus on includes the video update rate and flight information update rate. The former represents the frequency of understanding the environment, and the latter represents the control frequency of the UAV.

**Figure 13:**
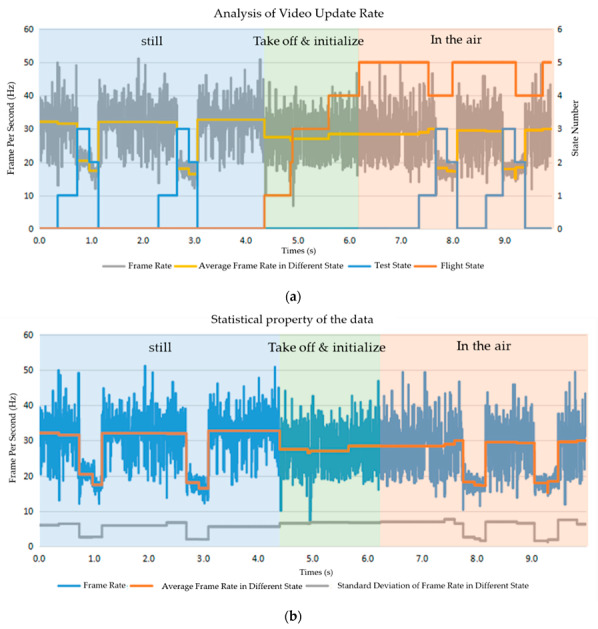
Video update rate analysis: (**a**) Analysis of video update rate; (**b**) Statistical properties of the data.

We utilize the frames per second (FPS) parameter to evaluate the video update. The frames (RGB images from the environment) are captured, processed (object detection and color blob detection), and displayed. Thus, this represents the frequency of understanding the environment. As shown in Figure 13, the average frame rate is around 30 Hz when none of the understanding method is carried out. As the UAV is moving in the air, the frame rate decreases a little due to the increasing computational cost of flight control. When the understanding method is fully running, the frame rate decreases to 18 Hz since the detection algorithm costs computational resources, although from the perspective of human feelings, the video is still smooth, and the real time property is enough.

**Figure 14:**
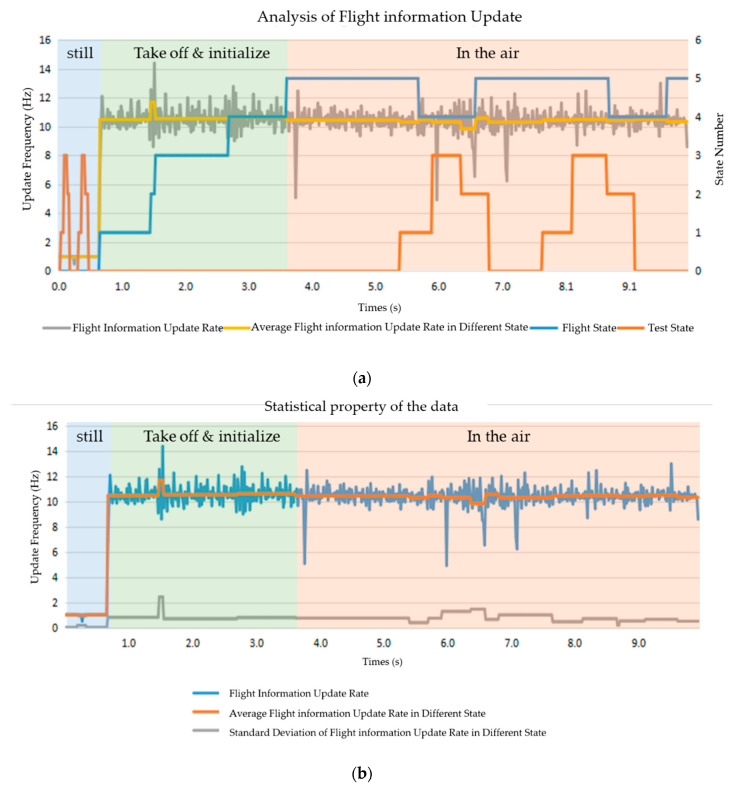
Flight information update analysis: (**a**) Analysis of flight information update rate; (**b**) Statistical properties of the data.

During a flight control loop, the smartphone sends the control command and the UAV returns the flight information. Thus, flight information update frequency represents the control frequency of the UAV. As shown in Figure 14, the flight information update rate is around 1 Hz when the UAV is on the ground with the motor powered off, and 12 Hz when the UAV is in the air. More frequent return of flight information makes the UAV safer in the air. From the figure, different test states have little influence on the flight information updates. That means the computational cost of the understanding method does not impede the updating of the flight information. Thus, the proposed understanding method runs with enough real time property in a safe way.

### 5.2. System Analysis

**Figure 15:**
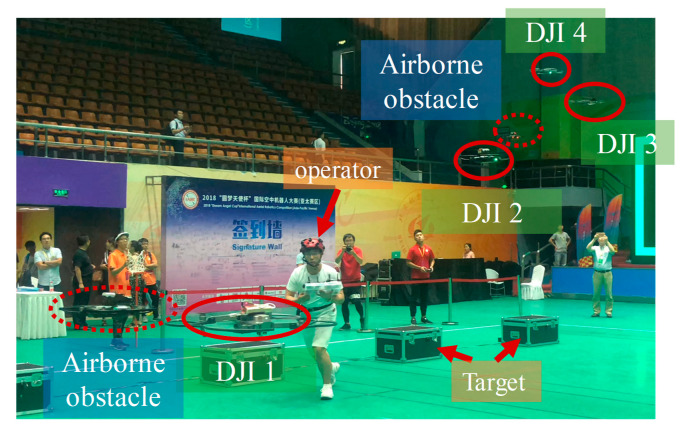
Snapshot of the IARC 2018 [33].

**Figure 16:**
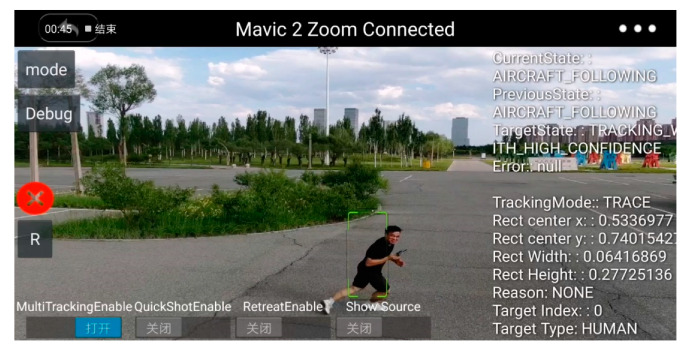
Outdoor estimation of the system.

With this method, we have participated in the MISSION 8 of the International Aerial Robotics Competition (IARC), 2018. The system won the first prize and the Best Man-Machine Team award in the competition, which also, to some extent, is proof of the practical value of the proposed method. As shown in Figure 15, DJI 1 to DJI 4 and the operator are the Man-UAV system. In the competition, the teams should design a Man-UAV system to find four target boxes, figure out the key of the lock to the target with the help of UAVs, and get out of the area with the items in the boxes within 8 min. During the competition, there are four aircraft that represent the enemy to trouble the system. Basically, the understanding method works well, while the performance still should be promoted. This competition relays on serval aspects of the system such as agile control to pierce through, the strategy, and the communication link. The discussion would be showed in next section. The system is tested outdoor as shown in Figure 16, and the detection method works. The large object could be detected successfully.

#### 5.2.1. Generality

This method is implemented in Java and deployed on the Android operating system, which ensures the generality. The method can run on all the embedded systems with compatible versions of the Android operating system. As for the UAV, all of the UAVs from DJI are compatible with the DJI Software Development Kit (SDK, designed by DJI) utilized in our program, which also ensures the generality.

#### 5.2.2. Transportability

It is easy to deploy the method onto another Android platform by installing an “*apk*“ file. When using a different size and type of UAV in different tasks, the operator could just connect the smartphone with the corresponding remote controller of the UAV. In the experiment, the method was transported successfully from a DJI Mavic Pro into a DJI Mavic Air, and from a Huawei Mate 10 pro into a Huawei Honor 6.

#### 5.2.3. Scalability

There are two update aspects. First, with the development of computer vision, the deep learning methods of understanding the environment are promoted day by day. In order to update the machine learning method proposed in this paper with newer methods, the approach is implementing the state-of-the-art methods with the TensorFlow architecture, then converting the model into a light version, and finally, replacing the light version model used in the program with the new one. This study successfully updated the object detection model from YOLO v3 to SSD-MobileNet. Second, the method proposed in this paper detects 80 common objects, while some scenarios requires the capability to detect special objects. To do that, one must train the new model with a special database, convert the model into a light version, and then replace the light version used in the program with the new one.

#### 5.2.4. Interactivity

**Figure 17:**
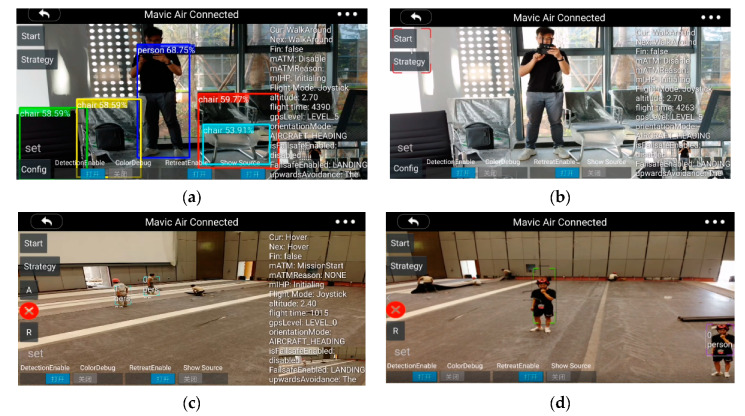
Screenshot of the smartphone: (**a**) object detection algorithm enabled; (**b**) object detection disabled; (**c**) detect the man with a red helmet and mark him with a purple rectangle; (**d**) detect and track the man with a red helmet.

The screenshot of the experiment is illustrated in Figure 17. The Figure 17a,b are taken when the UAV is connected to the computer for the semi-physical simulation, and Figure 17c,d are taken when the UAV is in the air. Figure 17a demonstrates the object detection works well. Figure 17d demonstrates the task, detecting and tracking the man with the red helmet, is completed successfully. As discussed above, the video is smooth enough for the operator.

## 6. Discussion

Compared with the proposal of Zhou et al. [10], our method is an online method and makes full use of the extremely fast properties of YOLO v3. The detection result could enhance the performance of an UAV, like alarming the operator after detecting the target, triggering the next mission, and participating in the decision making. This makes the UAV more intelligent, compared with the method proposed by Gao et al. [22]. The generic object detection capability enlarges the application scope of our method, compared with the work presented by Hummel et al. [22]. Clearly, the computational performance of hardware, especially smartphones, is increasing every year. Smartphones are more accessible than an embedded system with ROS. Therefore, our method has better generality and scalability than the method proposed by Gao et al. [22].

The experiments include two parts: semi-physical simulation and prototype experiments. Both are for the evaluation of the machine learning method’s capability to sense an unknown environment. The information from the environment could be represented by various aspects, including the object category, location of the object in the image, and color blob. This paper takes a further step on fusion of the representation at the action level for decision making and successful implementation. The task of detecting and tracking the man with a red helmet demonstrates the contribution. The method strikes a balance between high performance and the properties of generality, transportability, scalability, and interactivity. The real-time performance is enough, as tested by the task. The implementation of the method in this paper utilizes the multithreading mechanism to speed it up. It plays a major role in embedded systems with limited computational resources.

In the competition, we have tried to collect four videos from four UAVs and display them on one screen. However, this breaks down the system. There might be three reasons for this. Firstly, the video resolution is 1080 p, which could be decreased for high throughput; secondly, a different communication protocol could be tried like User Datagram Protocol (UDP) which has a higher refresh rate than TCP/IP; thirdly, the images could be processed in a distributed way.

There are two limitations of our method. The mechanical limitation of is our method is that the system works in the visible spectrum and depends on the weather conditions. The functional limitation of our method lies in that we can only recognize 80 categories of objects in an unknown environment. In the future, we plan to utilize multi-sensor data and more advanced computer vision algorithms to sense more information of the unknown environment.

Intelligent UAVs could perform object detection and target tracking so that they can auto-drive. There are several excellent methods, such as a model-free tracker [36] or a convolutional neural network (CNN)-based tracker [37,38]. To evaluate the performance of the tracker, Yu et al. [39] present a UAV dataset with 100 videos featuring approximately 2700 vehicles recorded under unconstrained conditions and 840 k manually annotated bounding boxes.

## 7. Conclusions

UAV swarms will play more and more important roles for humans in the future and detecting and identifying generic objects in unknown environments is the foundation for the high-level capability of UAV swarms. Through the semi-physical simulation, physical experiments and detection evaluation experiment, the designed system satisfies the need of generic object detection in real time, and strikes the balance between the high performance and the excellent properties of generality (can be used in various platform), transportability (easily deployed from one platform to another) and scalability (easily updated and maintained).

The method can be transported easily from one UAV to another and updated with a new state-of-the-art deep learning method in a convenient way. The computational speed and accuracy of algorithmd, especially the machine learning algorithm in the computer vision domain, progress every year, which reflect the importance of generality, transportability and scalability for the man-UAV system. This paper implements a closed cycle of images from capturing and processing to displaying and decision making. Semi-physical simulation and physical experiments evaluate the properties of the approach. The experiments demonstrate that the UAV could be armed with state-of-the-art computer vision algorithmw for the intelligence, and the real-time performance only slightly decreasew.

In our future work, we will extend the representation of the environment which could be sensed by the UAV. With these representations, the UAV would be more and more intelligent. More importantly, the higher level of function (post-processing of the detection) should be investigated, like navigating based on generic object detection capability.

## Figures and Tables

**Table 1:** Overview of the testbed.

Model	Size, Weight	Sensors	MSED ^1^	Lifetime	Video
**Mavic Pro**	83 × 83 × 198 mm, 734 g (with battery)	Gyroscope, accelerometer, binocular system, camera with gimbals	FCC: 7000 m; CE: 4000 m; SRRC: 4000 m	27 min, 3830 mAh	720 p @ 30 fps 1080 p @ 30 fps
**Mavic Air**	168 × 184 × 64 mm, 430 g (with battery)	Gyroscope, accelerometer, binocular system, camera with gimbals	FCC: 4000 m CE: 2000 m SRRC: 2000 m MIC: 2000 m	21 min, 2375 mAh	720 p @ 30 fps

^1^ Maximum signal effective distance (no interference, no shielding).

**Table 2:** States in the experiment.

State Number	Flight State	Test State
0	IDLE	None
1	Take off	Detect Color Blob
2	Initial Parameters	Detect Object
3	Maintain Channel	Detect Color Blob and Object
4	Hover	-
5	Walk Around	-

**Table 3:** COCO Dataset Detection Results.

	Smart Phone	AP ^2^	AP_50_^3^	AP_75_^4^	AP_S_^5^	AP_M_^6^	AP_L_^7^
YOLOv2 ^1^	-	21.6	44.0	19.2	5.0	22.4	35.5
SSD513 ^1^	-	31.2	50.4	33.3	10.2	34.5	49.8
DSSD513 ^1^	-	33.2	53.3	35.2	13.0	35.4	51.1
RetinaNET ^1^	-	**40.8**	**61.1**	**44.1**	**24.1**	**44.2**	**51.2**
YOLOv3 (608 × 608)^1^	-	33.0	57.9	34.4	18.3	35.4	41.9
Ours (320 × 320)	YES	18.6	30.5	19.9	0.07	14.6	46.8

^1^ The detection results are cited from Redmon et al. [21]. ^2^ AP: Average Precision at IoU = 0.50:0.05:0.95 primary challenge metric); ^3^ AP_50_: AP at IoU = 0.50 (PASCAL VOC metric); ^4^ AP_75_: AP at IoU = 0.75 (strict metric); ^5^ AP_S_: AP for small objects: area < 32^2^; ^6^ AP_M_: AP for medium objects: 32^2^ < area < 96^2^; ^7^ AP_L_: AP for large objects: area > 96^2^.

## Appendix A

The 80 classes that could be detected successfully are listed as follows: person, bicycle, car, motorcycle, airplane, bus, train, truck, boat, traffic light, fire hydrant, stop sign, parking meter, bench, bird, cat, dog, horse, sheep, cow, elephant, bear, zebra, giraffe, backpack, umbrella, handbag, tie, suitcase, frisbee, skis, snowboard, sports ball, kite, baseball bat, baseball glove, skateboard, surfboard, tennis racket, bottle, wine glass, cup, fork, knife, spoon, bowl, banana, apple, sandwich, orange, broccoli, carrot, hot dog, pizza, donut, cake, chair, couch, potted plant, bed, dining table, toilet, tv, laptop, mouse, remote, keyboard, cell phone, microwave, oven, toaster, sink, refrigerator, book, clock, vase, scissors, teddy bear, hair drier, toothbrush.
